# Bile Goes Viral

**DOI:** 10.3390/v13060998

**Published:** 2021-05-27

**Authors:** Victoria R. Tenge, Kosuke Murakami, Wilhelm Salmen, Shih-Ching Lin, Sue E. Crawford, Frederick H. Neill, B. V. Venkataram Prasad, Robert L. Atmar, Mary K. Estes

**Affiliations:** 1 Department of Molecular Virology and Microbiology, Baylor College of Medicine, Houston, TX 77030, USA; victoria.tenge@bcm.edu (V.R.T.); Wilhelm.Salmen@bcm.edu (W.S.); Shih-Ching.Lin@alumni.bcm.edu (S.-C.L.); crawford@bcm.edu (S.E.C.); Frederick.Neill@bcm.edu (F.H.N.); vprasad@bcm.edu (B.V.V.P.); Robert.Atmar@bcm.edu (R.L.A.); 2 Department of Virology II, National Institute of Infectious Diseases, Musashi-Murayama, Tokyo 208-0011, Japan; ko-mura@niid.go.jp; 3 Verna and Marrs McLean Department of Biochemistry and Molecular Biology, Baylor College of Medicine, Houston, TX 77030, USA; 4 Department of Medicine, Baylor College of Medicine, Houston, TX 77030, USA

**Keywords:** noroviruses, hepatitis viruses, bile acids, gastrointestinal infection

## Abstract

Laboratory cultivation of viruses is critical for determining requirements for viral replication, developing detection methods, identifying drug targets, and developing antivirals. Several viruses have a history of recalcitrance towards robust replication in laboratory cell lines, including human noroviruses and hepatitis B and C viruses. These viruses have tropism for tissue components of the enterohepatic circulation system: the intestine and liver, respectively. The purpose of this review is to discuss how key enterohepatic signaling molecules, bile acids (BAs), and BA receptors are involved in the replication of these viruses and how manipulation of these factors was useful in the development and/or optimization of culture systems for these viruses. BAs have replication-promoting activities through several key mechanisms: (1) affecting cellular uptake, membrane lipid composition, and endocytic acidification; (2) directly interacting with viral capsids to influence binding to cells; and (3) modulating the innate immune response. Additionally, expression of the Na^+^-taurocholate cotransporting polypeptide BA receptor in continuous liver cell lines is critical for hepatitis B virus entry and robust replication in laboratory culture. Viruses are capable of hijacking normal cellular functions, and understanding the role of BAs and BA receptors, components of the enterohepatic system, is valuable for expanding our knowledge on the mechanisms of norovirus and hepatitis B and C virus replication.

## 1. Introduction

Viruses are uniquely fascinating and challenging to study because they are obligate intracellular pathogens that often evolve a tropism not only for their specific hosts but for specific tissue and cell types. Molecular and physical techniques or animal models can be used to identify, detect, and study viral pathogenesis. However, cell culture is ideal for larger scale propagation of viruses, detailed studies of the mechanisms of viral replication, and studying the efficacy of antibodies and antivirals. Cell lines are often easy to genetically manipulate—a property that can be used to determine host factors required for infection. Several important human viruses, including hepatitis viruses and caliciviruses, remain difficult to cultivate despite advances in cell culture technology. Understanding cellular tropism and host factors was critical for the development of efficient systems to propagate these viruses. For the cultivation of hepatitis B virus (HBV) and human noroviruses (HuNoVs) that infect the liver and the intestine, respectively, bile components including bile acids (BAs) and BA receptors are now known to be key for successful replication [1,2]. BAs also influence infection of hepatitis C virus (HCV) and murine norovirus (MNV) through modulation of the innate immune response [3,4].

**Figure 1:**
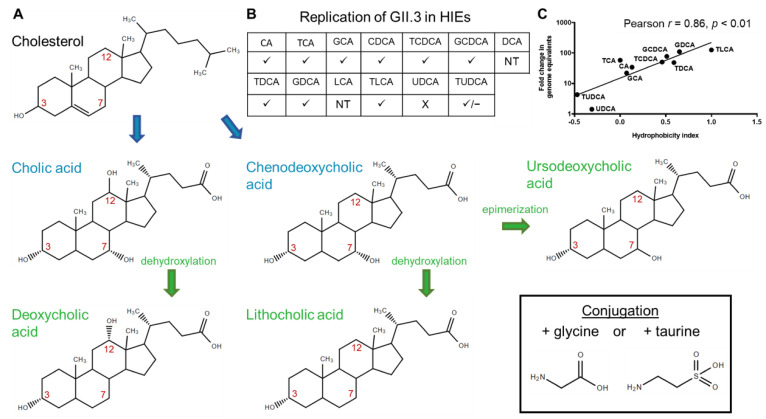
Bile acid pathways. (**A**) Primary bile acids (blue) are synthesized from cholesterol in the liver. Bacteria in the intestine convert primary bile acids to secondary bile acids (green) through dehydroxylation or epimerization. Carbons 3, 7, and 12 are indicated in red. Bile acids can be conjugated to glycine or taurine (box). (**B**) The effect of bile acids tested individually on HuNoV GII.3 infection of enteroids [1]. ✓ = permitted infection, X = did not permit infection, ✓/– = intermediate effect, NT = not tested at 500 µM due to cytotoxic effects (derived from Murakami et al., 2020 [1]). (**C**) Fold change in GII.3 genome equivalents correlates with the hydrophobicity indices of the bile acid used during infection. The solid line depicts the best-fit linear regression (*R*^2^ = 0.73, *p* < 0.01). The Pearson correlation coefficient (*r*) and *p* value (*p*) are noted (from Murakami et al., 2020 [1]).

Bile is a complex biologic mixture designed to aid in the digestion and absorption of fats and other nutrients [5]. Liver hepatocytes generate bile, which transits through the canaliculi to bile ducts. Bile flows through the hepatic duct and can be directed through the cystic duct into the gallbladder where it is concentrated and stored until secretion through the common bile duct into the upper small intestine (duodenum) during a meal [6,7,8]. Major components of bile include cholesterol, other lipids, bilirubin, and BAs — the primary focus of this review. In the liver, several enzymes are present to convert cholesterol to primary BAs including cholesterol 7α-hydroxylase that adds a hydroxyl group at carbon 7 required to form two primary BAs, cholic acid (CA) and chenodeoxycholic acid (CDCA) [9]. Sterol 12α-hydroxylase places an additional hydroxyl group at carbon 12 required to form CA [9]. Cholesterol and BAs all have a hydroxyl group at carbon 3. In the distal small intestine and colon, bacterial enzymes convert these to secondary BAs. The secondary BAs include deoxycholic acid (DCA) and lithocholic acid (LCA) formed by dehydroxylation of CA and CDCA, respectively, at carbon 7 [10]. Ursodeoxycholic acid (UDCA) is formed by epimerization of the 7α-hydroxyl group of CDCA carbon 7 to a 7β-hydroxyl group [8,11]. However, only low levels of UDCA are present due to gut bacteria-mediated conversion of UDCA to LCA [12,13]. BAs can be conjugated to glycine or taurine forming GCA, TCA, GCDCA, TCDCA, GDCA, TDCA, GLCA, TLCA, GUDCA, and TUDCA (Figure 1A). Conjugation reduces passive reabsorption into cells, and most BAs are actively reabsorbed in the ileum by the apical sodium-dependent bile acid transporter (ASBT) [10]. In the colon, secondary BAs that are deconjugated are passively reabsorbed [14]. BAs are subsequently transported into hepatocytes by Na^+^-taurocholate cotransporting polypeptide (NTCP) for recycling to the liver up to 4–12 times per day with the recycling pool containing primarily CA, CDCA, DCA, and trace amounts of LCA [10]. The cytosolic BA sensor, farnesoid X receptor (FXR) controls BA homeostasis through subsequent signaling pathways and has immunomodulatory activities [8,11,15,16,17]. BA receptors are summarized in Table 1.

**Figure 2:**
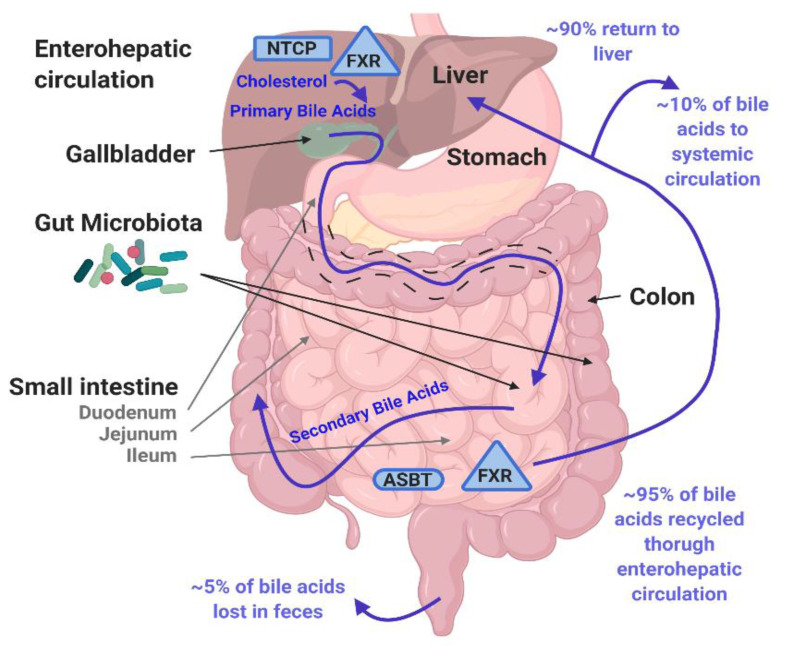
Enterohepatic circulation. Tissue expression locations of BA transporters ASBT (oval) and NTCP (rectangle) and BA binding protein FXR (triangle) discussed in this review are indicated. BAs are generated in the liver from cholesterol, stored in the gallbladder and then enter the duodenum in the small intestine. The gut microbiota in the small intestine and colon convert primary BAs into secondary BAs. Most (95%) BAs are actively reabsorbed in the ileum or passively reabsorbed in the jejunum and colon and recycled through enterohepatic circulation. Created with BioRender.com, accessed on 9 March 2021.

This process is termed enterohepatic circulation (Figure 2). Local total BA concentration in the lumen of the intestine can vary depending on whether an individual is in a fasted or post-meal state but can reach low millimolar concentrations [13,18,19,20,21]. Viruses that infect the liver and intestine encounter bile components in the extracellular milieu and intracellularly; therefore, it is not surprising that these pathogens have evolved mechanisms to co-opt BAs and/or BA-mediated cellular effects for their replication in host cells. This review focuses on mechanisms by which BAs regulate entry and infection of viruses that infect the liver and intestine and, in turn, further understanding of BA activities in the gastrointestinal system.

## 2. Norovirus Entry Mechanisms and Bile Acids

### 2.1. Bile Acids Mediate Norovirus Infection through the Activity of the Acid Sphingomyelinase Enzyme

HuNoVs are the leading cause of foodborne, epidemic, and acute gastroenteritis worldwide [22,23,24,25,26,27]. Some individuals who are immunocompromised (e.g., transplant, cancer, or common variable immunodeficient patients) can develop chronic HuNoV infection. In addition to health consequences, the economic cost of HuNoV is vast—an estimated $60 billion annually in direct healthcare costs and indirect loss of productivity costs [28]. HuNoV was first identified as the viral causative agent of a 1968 outbreak of gastroenteritis disease in Norwalk, Ohio by immune electron microscopy in 1972 [29,30]. For nearly five decades after their discovery, despite numerous attempts to cultivate these viruses in established continuous cell lines or 3D cell culture models, no system successfully and reproducibly permitted HuNoV replication [31,32,33,34,35,36,37,38].

**Figure 3:**
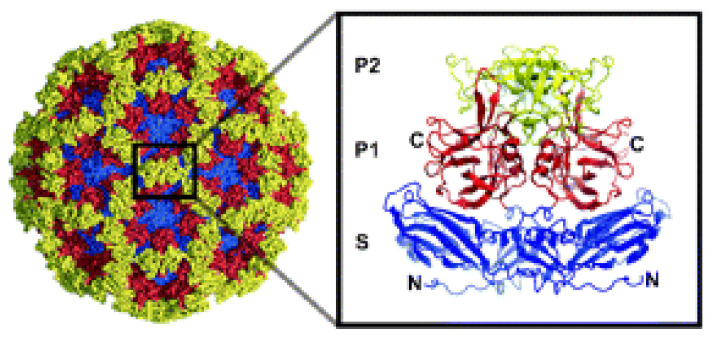
Norwalk (GI.1) VLP structure. The structure of a GI.1 VLP (Adapted from Choi et al. [43] copyright (2008) National Academy of Sciences). Inset: VP1 dimer. The VP1 dimer is organized into the shell domain (S, blue) and P-domain (P), which is further divided into P1 (red) and P2 (yellow). G1.1 strains that replicate in the HIE system are bile/BA-dependent.

HuNoVs are non-enveloped single-stranded positive-sense RNA viruses in the *Caliciviridae* family. The capsid contains viral structural proteins VP1 and VP2. VP1 forms the capsid structure and is divided into two domains: a protruding domain (P-domain) and a shell domain (Figure 3). Virus-like particles (VLPs) form through self-assembly of VP1 + VP2 or VP1 alone. Despite the initial lack of a cell culture system for infectious HuNoV, studies with VLPs identified a group of glycans called the histo-blood group antigens (HBGAs) as initial binding factors for interactions with intestinal cells and a subset of red blood cells [39,40,41,42]. HuNoVs bind HBGAs or related human milk oligosaccharides through the outer P-domain on the viral capsid [43,44,45], consistent with that domain being the site that initially interacts with cells. VLPs were shown to bind to HBGAs found on intestinal cells only from secretor-positive individuals [40] and subsequent epidemiological and volunteer studies showed that susceptibility for infection only occurs in individuals who express a fucosyltransferase 2 gene to produce a functional fucosyltransferase 2 that adds fucose to glycan chains on proteins or lipids. People with this enzyme are called secretor-positive individuals and they are susceptible to infection with many HuNoV strains [46,47,48]. HBGA binding shows strain-specificity as the GII.4 strain VA387 major contact is to α-fucose and another strain, GI.1 Norwalk virus, has major contacts with α-N-acetyl galactosamine or β-galactose of HBGA molecules [49]. However, the presence of HBGAs, though required for infection, is not sufficient for infection of typical established laboratory cell lines initially made from cancer cells [50].

**Figure 4:**
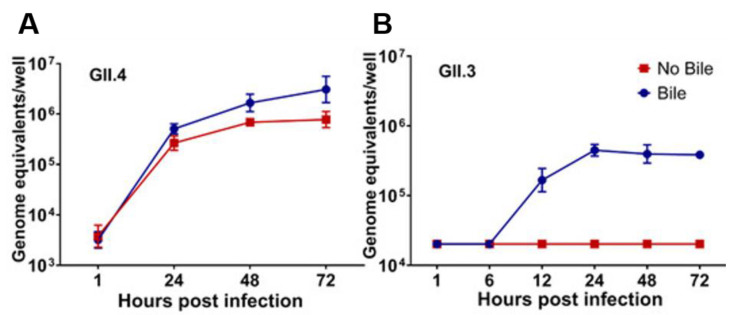
GII.3 but not GII.4 HuNoV infection requires bile. Virus growth curves show enhanced RNA replication of GII.4 in the presence of human bile (**A**). Replication of GII.3 required the addition of human bile to cultures (**B**). Error bars denote standard deviation (from [51]. Reprinted with permission from AAAS).

**Figure 5:**
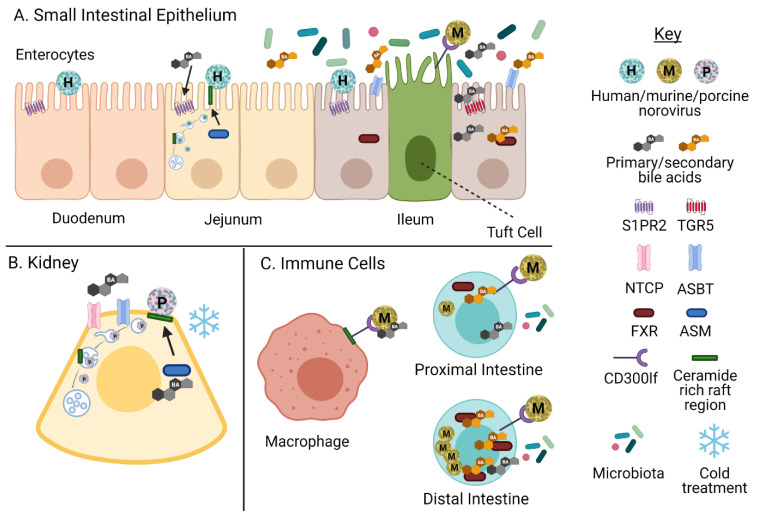
Models for bile acid-mediated infection by noroviruses in different cell types. (**A**) Bile-dependent and -independent human noroviruses infect duodenal, jejunal, and ileal HIEs. The mechanism of GII.3 BA mediated infection in HIEs is well characterized in jejunal HIEs [1]. MNV infects tuft cells of the mouse ileum [54]. (**B**) PoSaV infects porcine kidney cells in a BA-dependent manner described below. (**C**) MNV infects immune cells (right) [55,56]. BAs influence replication of MNV in different regions of the intestine (left). Adapted by permission from Springer Nature: Springer Nature, Nature Microbiology, from ref. [4], copyright (2019). Created with BioRender.com, accessed on 12 March 2021.

In 2016, we established an intestinal epithelial replication system for HuNoV cultivation in human intestinal enteroids (HIEs), also called organoids [51,52]. Initial studies in HIEs show the globally dominant GII.4 strain of HuNoV can replicate in the absence of bile but three other strains tested (GI.1, GII.3, and GII.17) required the addition of bile (Figure 4) [51]. This HuNoV replication system provides a platform for investigating the role of bile and components of enterohepatic communication in regulating viral infection. Initial attempts to characterize the critical factor in bile required for HuNoV replication were unable to identify a specific component but indicated that the active component was not a protein [51]. Subsequent testing of a panel of BAs individually at sub-micellar concentrations for their ability to permit GII.3 replication in jejunal HIEs established that BA-mediated replication of GII.3 correlates with the hydrophobicity of the BA (Figure 1B,C) [1]. The secondary BA, UDCA fails to permit GII.3 infection [1,52]. Its taurine conjugate, TUDCA, permits low levels (lower fold-change compared to infection with whole bile) but inconsistent infection. GCDCA, a highly effective BA that lacked cytotoxicity, used in time-course experiments showed the replication-promoting effect occurs at early time points through an effect on the cells [1]. Subsequently, it was determined that GI.1, GII.1, GII.6, and GII.17 replicate in HIEs only with the addition of GCDCA during infection [53]. The cellular consequences of GCDCA treatment are multifold: GCDCA treatment of HIEs leads to increased cellular uptake of a fluorescent membrane probe through the BA receptor sphingosine-1-phosphate receptor 2 (S1PR2) followed by GII.3 VLP endosomal uptake, endosomal acidification, and generation of ceramide at the apical cell surface through a critical enzyme, acid sphingomyelinase (ASM) [1]. Inhibitors of S1PR2, endosomal acidification, and ASM reduce GII.3 infection levels, while exogenous addition of ceramide alone permits modest virus replication [1]. Based on these data, we propose a model for jejunal GCDCA-dependent GII.3 infection where GCDCA acts through S1PR2 and ASM, leading to the development of ceramide-rich regions on the cell surface that contains the unknown receptor for GII.3 HuNoV. Once bound to its receptor, the virus is internalized through BA-mediated cellular uptake and transits through an endosomal pathway prior to escape of the virion or genome to the cytosol (Figure 5A). GII.3 can also infect the duodenal and ileal segments of the small intestine in the presence of GCDCA but whether the mechanism of BA stimulation of virus replication is the same as in the jejunum remains to be studied. Colonic cultures treated with secondary BAs have not supported HuNoV replication even when these cultures are produced from the same secretor-positive individuals whose duodenal and ileal cultures support virus replication in the presence of GCDCA [53].

Other viruses in the *Caliciviridae* family [porcine sapovirus (PoSaV), feline calicivirus (FCV) and MNV] had earlier established culture systems, defined cellular receptors and are often used as surrogates for HuNoV infection [54,55,56,57,58,59,60,61,62,63,64,65]. Our studies with HuNoV are reminiscent of early data showing the importance of BAs for infection of another enteric calicivirus, PoSaV, and for subsequent studies showing BAs have a role in MNV infection (Figure 5B,C). GCDCA-mediated replication of PoSaV in porcine kidney cells is the most similar surrogate model for GII.3 HuNoV replication. PoSaV was originally shown to replicate in porcine kidney (LLC-PK) cells in the presence of intestinal contents from gnotobiotic pigs [57,58]. Later, some individual BAs (CA, CDCA, GCA, GCDCA, TCDCA, DCA, LCA, GDCA, and TLCA) were found to permit PoSaV infection [66]. These individual BAs overlap with BAs that permit GII.3 HuNoV replication in HIEs, and similar to GII.3 infection, UDCA and TUDCA do not permit PoSaV infection (Figure 1B). Similar to the entry effects of GCDCA on GII.3 HuNoV infection, a time course of GCDCA addition during PoSaV infection shows the greatest effect on replication is if GCDCA is present during the 1 h inoculation period, and PoSaV will no longer replicate if GCDCA is added as late as 4 h post-inoculation [67]. The addition of an endosomal acidification inhibitor, chloroquine, during PoSaV infection negates GCDCA-mediated replication, and PoSaV becomes trapped in Rab7-positive late endosomes, demonstrating the importance of endosomal acidification on endosomal escape for virus infection [67]. In the absence of GCDCA, PoSaV also becomes trapped in Rab7-positive compartments [68]. In the human cultivation system treatment of jejunal HIEs with GCDCA alone for 1 h increases the numbers of Rab7 compartments [1]. When BA-induced acidification of HIEs is inhibited with bafilomycin A1 and ammonium chloride, the number of Lysotracker-stained acidic compartments and GII.3 infection are reduced [1]. Future studies of GII.3 late endosomal colocalization in the presence of pH inhibitors and GCDCA are necessary to determine if GII.3 becomes trapped in Rab7-positive late endosomal compartments. FCV and MNV both require endosomal acidification during entry into Crandell-Rees feline kidney (CRFK) and RAW267.4 mouse macrophage cells, respectively, despite their ability to replicate in the absence of BA [67]. Together, these studies highlight a common role of acidification in calicivirus entry into different cell types that is shared by both human and animal viruses, but MNV and FCV may be capable of initiating acidification without the addition of BA. Recently, a replication system was developed for an additional previously noncultivatable genus of *Caliciviridae*, the human sapoviruses (HuSaVs). HuSaV strains GI.1 and GII.3 were shown to replicate in NEC8 (human testis) and HuTu80 (human duodenum) cell lines [69]. Additionally, GI.2 HuSaV replicates in HuTu80 cells. Replication of HuSaVs requires the presence of bile or BA and the most efficient BAs tested were GCA and GCDCA. The mechanism of BA-mediated HuSaV replication and its effect on endosomal acidification and ceramide production in NEC8 and HuTu80 cells needs further elucidation to determine whether HuSaV also requires these cellular pathways.

A serendipitous discovery was that during inoculation of LLC-PK cells with PoSaV in the cold (1 h binding incubation was at 4 °C) the virus was able to replicate in the control wells without GCDCA and the virus could escape endosomes (Figure 5B, snowflake) [70]. Based on observations that the membrane lipid ceramide can influence membrane perturbations [71,72,73] and that ceramide can be generated after cell stress, the authors looked for ceramide generation after GCDCA or cold treatment [70]. Ceramide is induced after cold treatment, and this induction is blocked by acid sphingomyelinase (ASM) inhibitors or siRNA targeting ASM [70]. FCV and MNV infections also induce ASM activity in CRFK or RAW267.4 cells, respectively, and as observed for GII.3 infection in HIEs, replication of all three surrogate viruses is blocked by ASM inhibition [70]. Moreover, the ASM inhibitors block the endosomal escape of PoSaV, FCV, and MNV [70]. In HIEs, ceramide generation after GCDCA treatment is detected at the apical surface rather than internally. Internal cellular ceramide can be difficult to detect by staining, and there is still the potential that ASM is active in endosomes while the virus is transiting through the endosomes and generating additional ceramide that would destabilize the endosome leading to GII.3 escape (Figure 5A). It remains to be determined if ASM inhibition reduces GII.3 infection solely by preventing apical ceramide generation or if inhibition of subsequent endosomal ceramide formation blocks GII.3 escape. Further studies with HuNoV strains that both require, or do not require BA for replication, should determine whether ASM inhibition leads to failure of the virus to escape the endosome. Cold pretreatment of HIEs did not lead to GII.3 HuNoV infection or replication in the absence of GCDCA (unpublished data), implying cold stress may not trigger ceramide generation in HIEs as it does in LLC-PK cell lines. Like MNV and FCV, GII.4 HuNoV does not require BA, but the infection is enhanced by BA and is blocked by ASM inhibition. Future experiments will determine if GII.4 VLPs alone might trigger ceramide generation.

Several other viruses use the ASM-ceramide pathway in infection: group B and C adenoviruses (AdV), measles virus, rhinovirus, Japanese encephalitis virus, and ebolavirus [74,75,76,77,78]. In the AdV model, a structural protein, protein VI, present in the capsid, triggers a membrane wounding response and ASM lysosomal exocytosis by mild membrane disruption followed by ceramide generation at the surface [74]. When AdV subsequently enters cells through endocytosis, acidification allows more protein VI to release from the capsid, and it can interact with ceramide in endosomal membranes to create pores for viral escape [74]. Both enteric and nonenteric AdVs have recently been shown to replicate in HIEs [79]. It would be interesting to determine if the ASM-mediated entry mechanism is also utilized by these AdVs in the HIE model and if ceramide generation can be detected at both the apical and endosomal membranes. Recently, FCV VP2 was shown to form a pore structure after virus engagement with its receptor [80]. An interesting speculation is that this VP2 pore may interact with ceramide in the endosomal membrane, which in combination with receptor engagement permits endosomal escape of the calicivirus RNA genome into the cell. The VP2 of FCV and HuNoVs are quite distinct in terms of size (106 amino acids and 208–268 amino acids long, respectively), and whether HuNoV VP2 performs a similar function, or whether either VP2 can bind to ceramide, remains to be determined.

### 2.2. Direct Binding of Bile Acids to Norovirus Capsids

**Figure 6:**
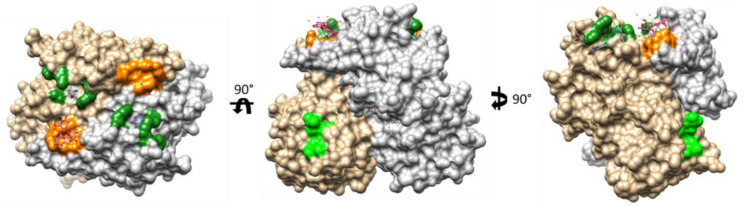
Bile acid binding sites on the GII.10 HuNoV P-domain. Monomers of the GII.10 P-domain dimer colored in grey and tan. Dark green: BA binding site identified by Kilic et al. with GCDCA complexed [81]. Light green: putative low affinity BA binding site reported by Creutznacher et al. [82]. Orange: HBGA binding site complex with 3-fucosyllactose (from Weichert et al., 2016) [45].

Structural studies suggest that some BAs can interact with the major capsid protein (VP1) of certain HuNoVs (Figure 6). By isothermal titration calorimetry (ITC), a GII.10 HuNoV VLP and the P-domain from GII.1, GII.10, and GII.19 were shown to interact with primary BAs [81]. Important to note, GI.1 and GII.4 VLPs as well as GI.1, GII.3, GII.4, and GII.17 P-domains were also tested but did not bind BA. These latter viruses are those that replicate in the HIE HuNoV cultivation system and whose replication either requires (GI.1, GII.3, GII.17) or is enhanced (GII.4) by BA treatment. As described above, for GII.3 BA-mediated infection, we have shown BA treatment of the cells and subsequent cellular effects mediate infection [1]. GII.1 replicates in the HIE cultivation system in media supplemented with BA but not in media alone [53]. Thus far, GII.10 virus does not replicate in the HIE system, and the porcine GII.19 virus has not been tested. A limitation is that our infection system uses stool filtrate virus acquired by collection of clinical samples and there are, in comparison to GII.4, relatively few GII.10 positive stools to test.

GII.1 is an interesting virus as it or its VLP initially did not bind to the HuNoV initial cellular binding factor (HBGAs), but subsequent studies show GII.1 VLPs treated with BA are able to bind porcine gastric mucin (PGM; contains HBGAs) and binding of GII.10 to porcine gastric mucin is also enhanced [81]. Using a similar PGM binding assay, we tested our available VLPs (Table 2) and confirmed that a GII.1 VLP is only able to bind to HBGA.

**Figure 7:**
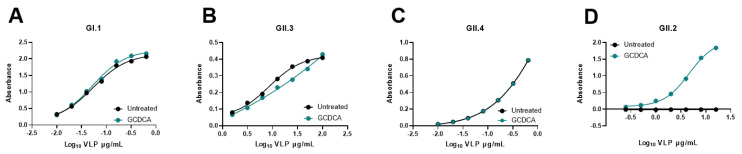
GCDCA enhances GII.2 VLP but not GI.1, GII.3, and GII.4 VLP binding to PGM. (**A**–**D**) VLPs were tested for their ability to bind to PGM (source of HBGAs) by ELISA in the presence or absence of 500 µM GCDCA. VLPs used are described in Table 1. Previously unpublished data (**A**,**C**,**D**). Derived from Murakami et al., 2020 (**B**) [1]. The PGM binding assays performed are described in Murakami et al., 2020 [1].

When 500 µM GCDCA is added to the assay. GI.1, GII.3, and GII.4 binding to PGM was unaffected by GCDCA (Figure 7A–C). Additionally, we found that GII.2 (Figure 7D) and GII.12 VLPs require the presence of GCDCA to bind PGM. Both GII.2 and GII.12 HuNoVs replicate in the presence of GCDCA but neither were tested in the absence of BA [53,83]. The replication-promoting effect of GCDCA on GII.2 replication may differ from GII.3 in that GCDCA binding to the GII.2 virus could solely be required for interactions with HBGA on the cell surface that trigger entry. However, further studies are necessary to test if cellular effects of GCDCA treatment that permit GII.2 replication are the same as those characterized and required for GII.3 replication. Additionally, GII.2, has been shown to infect a subset of individuals that have secretor-negative HBGA expression, and bile addition permits GII.2 VLP binding to saliva HBGAs from secretor-negative individuals [84]. Whether this results in replication in HIEs from secretor-negative individuals remains to be determined.

Saturation transfer difference-NMR (STD-NMR) low affinity (millimolar range) binding data have been used as another method to evaluate HuNoV VLP binding to BAs. The results indicate there is a binding interaction of GI.1, GII.4, GII.7, GII.10, and GII.17 HuNoV VLPs near the C terminus of the P-domain with primary BAs. This interaction site is not the same as the high affinity (low micromolar range) BA binding region at the top of P2 near the HBGA binding site as seen in the ITC experiments with P-domains of GII.1, GII.10, and GII.19 [82]. Of these, GI.1/GII.4/GII.7/GII.17 all infect HIEs and of which GI.1 and GII.17 strictly require BA [51,53]. However, it remains unknown whether BAs bind VLPs of GII.3, which is the virus strain we have performed mechanistic studies on the cellular effects of BA-mediated replication [1], analyzed by the same technique. Interestingly, this low affinity BA binding site does not affect the binding of the P-domains to HBGA, which suggests that BA binding may contribute to additional roles independent of HBGA binding [82]. Additional studies are needed to determine if the low-affinity direct interactions with BAs are biologically relevant during infection of GI.1 and GII.17 and if BA-mediated cellular requirements necessary for GII.3 infection are also critical for GI.1 and GII.17.

In addition to infection of RAW267.4 cells, MNV infects several other cell types including B cells, dendritic cells, neutrophils, myelomonocytic cells, and tuft cells based on the expression of the CD300lf receptor, innate immune signaling, and strain persistence [85]. BA binding to MNV differs from the reported GCDCA binding locations for HuNoVs; GCDCA and LCA bind to the MNV P-domain between the P1 and P2 subdomains at the P dimer interface with low micromolar affinity [86]. GCDCA increases MNV binding to cells and enhances infectivity [86]. MNV and GCDCA binding has a small positive effect on interaction with the MNV receptor, CD300lf, which is expressed on macrophages, dendritic cells, and tuft cells (Figure 5A, tuft cell and Figure 5C) [54,86]. Though the role of BA was not evaluated in a separate receptor study, CD300lf is required for infection of tuft cells by the persistent strain CR6 [85]. For the nonpersistent CW3 strain, infection of myelomonocytic cells is reduced by CD300lf disruption and viral levels detected during infection of other cell types (e.g., B cells) is unaffected [85]. Some norovirus VP1 structures show conformational flexibility and can exist in an open or a closed/compressed conformation [87]. GCDCA and TCA cause stabilization of the collapsed conformation of the MNV P-domain, allowing better interaction with the receptor [88,89]. The collapsed BA-interacting conformation of MNV P-domains is not compatible with binding of P-domain to the Fab of neutralizing antibody A6.2 and increasing BA concentrations prevent neutralization of MNV infection by antibodies 4f9.4, A6.2, and 2D3 in BV-2 cells [90]. These effects are likely specific to MNV because GII.4 HuNoV infection, which is also enhanced by GCDCA, does not require the CD300lf receptor [65]. However, capsid plasticity may still have a biological role in HuNoV infection as GII.3 P-domain dimers are shown to exist in both a resting and rising conformation [91]. Additionally, the HuNoV receptor(s) is yet to be identified. Once determined, structural studies with purified HuNoV or VLP in the context of receptor binding will reveal if BA-capsid binding promotes stabilization of P-domain conformations that favor receptor interactions. How BA binding influences HuNoV entry processes downstream of receptor binding (e.g., endosomal escape and genome release) and how BA simply enhances GII.4 replication also remains to be determined.

## 3. Bile Acids Influence FXR and Innate Immune Regulation to Promote Infection of Some Hepatitis Viruses and Caliciviruses

The gut–liver axis, mediated by BAs has an important role in not only nutrient digestion and absorption but also in epithelial cell proliferation, regulation of inflammation, and the interplay between the microbiota and host [10,13,92,93,94]. Several viruses, including hepatitis A–E, are hepatotropic and can lead to acute or chronic liver injury. Prolonged and chronic infection leads to the development of more severe liver disease, including fibrosis, cirrhosis, hepatocellular carcinoma, and death [95].

Hepatitis C virus (HCV) is an enveloped RNA virus in the Flaviviridae family and causes acute hepatitis. Some infected persons will clear the virus, but most (50–80%) develop chronic HCV, which over time causes adverse effects on the liver leading to hepatic fibrosis, cirrhosis, and hepatocellular carcinoma [96]. Combinations of antivirals can treat and clear chronic infection, but there is no vaccine [96]. Akin to the HuNoV field, research on HCV was restricted by a lack of cell culture system for many years [97]. About a decade after their discovery in 1989, a replicon system was developed by replacing regions of the viral genome encoding structural genes with selection markers. Replication of this system is most efficient in the liver cell line Huh7 [97]. After over another decade, multiple similar replication systems were developed using Huh7 cell line derivatives such as Huh7.5.1, which has a mutation in the innate immune sensor RIG-I permittingbetter replication. Further advancement was made with the discovery that an HCV full-length genome, from the JFH-1 (genotype 2a) isolate from a 32-year-old male patient with fulminant hepatitis, replicated efficiently when transfected into Huh7 cells [97,98,99,100]. JFH-1 virus generated after transfection infects Huh7 cells with low efficiency but infects with higher efficiency in Huh7.5.1 cells [99,101]. Virus in this culture system can be passaged but further optimization is necessary to understand the high variability in viral titer seen with replication of different virus strains and in different Huh7 lines, and to expand the number of isolates that can be cultured.

HCV genotype 1b subgenomic replicons have been used to probe factors that might enhance replication. The primary BA CDCA and secondary BA DCA enhance HCV RNA replication in a dose-dependent manner (20–100 µM) [3,102]. The secondary BA LCA is also effective at promoting HCV genotype 1b replication but UDCA and CA only have mild enhancing effects [102,103]. Glycine-conjugated BA GCDCA only promotes replication at the highest concentration tested (200 µM) [3,102]. Glycine and taurine conjugated BAs GDCA, TDCA, and TCDCA also fail to promote HCV genotype 1b replication [102]. Together, these data indicate that conjugated BAs are less effective at allowing for HCV replication. Conjugated BAs do not passively diffuse across the plasma membrane, and continuous liver cell lines, including Huh7, lack expression of BA transporters such as NTCP [2,8,104], suggesting an internal BA receptor that nonconjugated BAs can reach mediates the promoting effects for virus infection.

FXR, an intracellular BA sensor, detects internalized BAs and through downstream signaling pathways, regulates metabolism including BA and lipid homeostasis [105]. An FXR antagonist, Z-guggulsterone, and siRNA against FXR reduce the CDCA-mediated increase in replication of the HCV replicon [3,102]. However, replication of a genotype 2a HCV replicon is not enhanced by CDCA treatment nor inhibited by Z-guggulsterone, demonstrating strain-specific differences [102,103]. Later advances in HCV cultivation have allowed for replication of full genomes of both genotype 1b and 2a, and both are enhanced by CA and CDCA, indicating there may be additional BA effects on HCV genotype 1b and 2a during full genome replication. The importance of FXR has not been tested in this system [103]. Studies with PoSaV also report that BA-mediated replication in porcine kidney cells is inhibited by the FXR antagonist although the data are not shown [3].

Both HCV and Norwalk (GI.1 norovirus) replicon systems are inhibited when cells are incubated with exogenous interferon (IFN)-α or IFN-γ. Addition of BAs CDCA and DCA can partially rescue the IFN-mediated inhibition of HCV infection in a dose-dependent manner when co-incubated with IFNs [3]. Only the highest GCDCA and UDCA concentrations block the action of IFN [3]. FXR suppresses innate immune factors through trans-repression [17]; therefore, a potential explanation is that FXR activation by BAs that can passively enter cells suppresses HCV- or Norwalk-activated IFN pathways in the replicon systems. Replication of a GI.1 Norwalk virus replicon in HG23 liver cells is not enhanced by CDCA, DCA, UDCA, or GCDCA, which enhances HCV replication [3]. In contrast, DCA and CDCA rescue IFN-suppressed replication of the Norwalk replicon expressed in HG23 cells, but only when the highest concentration of each BA tested is preincubated with cells 24 h prior to IFN treatment. This might indicate maintenance of the Norwalk replicon in liver cells could be priming an immune response that is rapidly activated by IFN, and pretreatment with BA downregulates this. However, transfection of HEK 293FT cells with Norwalk GI.1 virus genomic RNA isolated from stool does not activate an innate response [106], although long-term maintenance of a viral replicon could have a stronger effect on activating innate responses. The effect of a BA-FXR-IFN suppression axis does not appear to be a driving factor in replication for human and porcine caliciviruses that replicate in epithelial cells (Figure 5A,B). An initial study with BA-mediated PoSaV replication in porcine kidney cells reported that the effect of BAs is through downregulation of signal transducer and activator of transcription 1 (STAT1) by BA [66]. However, later studies with PoSaV refuted this conclusion [107], and an alternative mechanism of BA-mediated replication through effects on acidification and ASM-generated ceramide was proposed (discussed above, Figure 5B) [67,70]. In the HIE GII.4 and GII.3 HuNoV cultivation system, exogenously added type I and III IFN inhibit replication, and the BA-dependent GII.3 strain is sensitive to endogenous IFN responses despite the presence of BA in the media during infection [108]. Agonists or antagonists of FXR do not affect GII.3 replication in HIEs, and BAs instead influence viral entry through interaction with the GPCR S1PR2 BA receptor (see Section 2.1 above) [1].

**Figure 8:**
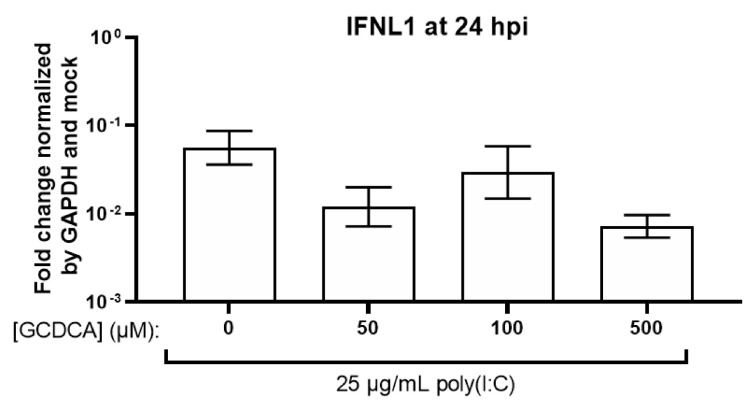
GCDCA in combination with poly(I:C) treatment of HIEs does not induce a greater type III IFN response. Jejunal HIEs were treated with 25 µg/mL poly(I:C) and increasing concentrations of GCDCA. At 24 h post treatment, mRNA levels of *IFNL1* (shown), *IFNL2*, and *IFNL3* were assayed. Previously unpublished data. Poly(I:C) treatment of HIEs and RT-qPCR for mRNA levels of type III IFNs performed as in Lin et al., 2020 [108].

MNV infection of intestinal cells appears to share the requirement for FXR-mediated downregulation of IFN responses. In the mouse proximal and distal small intestine, bacteria are reported to deconjugate BAs to remove glycine and taurine generating primary BAs and additional bacterial enzymes generate secondary BAs. In the mouse proximal small intestine, these modified BAs along with type III IFN (IFN-λ) restrict MNV infection [4]. In a mouse intestinal epithelial cell line (CMT-93) that is not permissive to MNV, poly(I:C), used as a surrogate for viral RNA, leads to some induction of IFN-λ. CMT-93 cells primed by a 12 h pretreatment of the BAs CDCA and DCA but not CA or LCA express significantly more IFN-λ upon treatment with poly(I:C) compared to unprimed poly(I:C) treatment [4]. An epithelial cell line engineered to express the MNV receptor, CD300lf, has significant induction of IFN-λ upon MNV infection after priming with DCA compared to unprimed infection [4]. CDCA priming also increased IFN-λ but in a non-significant manner [4]. These data are proposed to indicate that bacterial modified BAs found in the small intestine restrict MNV replication in the proximal small intestine by priming cells for a type III IFN response (Figure 5C, right) [4]. By contrast, in the distal small intestine, there is a greater expression of FXR, which is activated by BAs. CMT-93 cells treated with an FXR agonist, GW4064, show a dose-dependent decrease in IFN-λ [4]. This implies that in the mouse distal small intestine, shown to have much higher levels of FXR mRNA compared to the proximal small intestine, BA presence primes the intestine for an IFN response upon infection and activates FXR. However, concurrently, FXR immune-modulating activity negates the immune response permitting MNV replication (Figure 5C, right) [4]. The BA effect on IFN-λ and the importance of FXR differ in the HIE model system. Jejunal HIEs treated with both GCDCA and poly(I:C) at the same time show no differences in IFN-λ induction in the presence of GCDCA compared to poly(I:C) alone (Figure 8). Pretreatment with GCDCA or other BAs prior to addition of poly(I:C) was not tested. GII.3 can replicate through BA-mediated mechanisms in enterocytes from duodenal, jejunal, and ileal HIEs, and in jejunal HIEs, FXR is not involved [1]. The importance of FXR during GII.3 infection of duodenal or ileal segments remains to be tested. Together, data from the HCV, PoSaV, HuNoV, and MNV replication systems indicate that BA mechanisms that regulate replication are virus- and cell-type-specific. While mechanisms of action may differ, these human and animal viruses serve as new models to further understand new aspects of BA signaling and biology in the intestine and liver.

## 4. Bile Acids Alter Forms of HAV and HEV Virus

Bile acids have a novel role in the biology of hepatitis A and hepatitis E viruses (HAV and HEV). These phylogenetically unrelated non-enveloped viruses are transmitted through a fecal–oral route. HAV and HEV replicate primarily in the liver and pass through the biliary canaliculi with high concentrations of bile acids prior to release into the duodenum. Studies of HAV first reported a new paradigm, wherein quasi-enveloped particles (eHAV) are found to be released from cells cloaked in host-derived membranes. While these quasi-enveloped virions are the only particle type found circulating in blood during infection, only nonenveloped virions are shed in feces [109]. Further studies showed high concentrations of BAs (24 mM CDCA and 93 or 930 mM TCA) can convert eHAV to nonenveloped virions while virions in bile contained both particle types [110]. These studies indicate nonenveloped virions shed in feces are derived from eHAV released across the canalicular membrane and are stripped of membranes by the detergent action of BAs within the proximal biliary canaliculus. Subsequent studies showed HEV also exists as naked and quasi-enveloped particles [111]. Bile is also thought to degrade the eHEV membrane, resulting in the non-enveloped HEV in feces [112]. These novel membrane-cloaked virus particles for both HAV and HEV are infectious in cell culture yet viral antigens are masked due to the engulfing membrane and they are resistant to neutralizing antibodies, stimulating many new studies about how they influence viral pathogenesis. In vivo experiments with human liver chimeric mice and cell culture-derived HEV required the virus-containing cell supernatants to be treated with DCA and trypsin for successful infection [113]. Buoyant density analysis shows that an untreated supernatant virus is similar in profile to a virus found in mouse plasma and the supernatant virus treated with DCA and trypsin had two density populations [113]. This result is consistent with loss of the envelope as HEV particles encounter bile prior to being shed in feces. Whether HEV has encountered bile components (feces virus) or not (serum virus) may affect transmission between hosts and should be further investigated.

Recent evidence indicates norovirus particles can be shed in the stool as membrane-cloaked enveloped vesicles. Beads capable of binding to enveloped vesicles are able to isolate vesicles positive for HuNoV VP1 and markers of multivesicular body-derived (MVB) exosomes from the stool of infected patients and these vesicles contain one to five particles [114]. Similarly, MNV is found in isolated extracellular MVB-derived exosomes from infected RAW267.4 cells [114]. Both HuNoV and MNV containing exosomes appear to be infectious in HIEs and RAW267.4 cells, respectively [114]. Norovirus enveloped particles produced and released into the small intestine encounter lower levels of BAs than those found to convert eHAV or eHEV into naked particles, so noroviruses are unlikely to be affected in a similar manner. Therefore, what role BAs may play in infection, receptor binding, or HBGA binding in exosome-contained norovirus infection remains to be determined.

## 5. Role of the BA Receptor NTCP in HBV and Enteric Virus Infection

The hepatitis B virus (HBV) from the *Hepadnaviridae* family is another cause of liver disease in humans. HBV is associated with a satellite virus, hepatitis D (HDV) that requires the acquisition of HBV surface antigen (S) during assembly to subsequently form viral particles that can then enter cells [115]. There is a vaccine for HBV, but according to the CDC, over 350 million people are infected with HBV worldwide and although 95% of infected adults will never develop chronic infection, 25–50% of infected children will go on to have chronic HBV. Chronically-infected individuals suffer from liver diseases caused by infection including cirrhosis, hepatocellular carcinoma, and liver failure that lead to death [2]. Development of antivirals is limited due to the previous lack of laboratory cell lines that can be infected and recapitulate the full infectious life cycle [2,116]. HBV is an enveloped virus with three forms of a viral surface protein: small (S), middle (M) that contains the S domain and pre-S2 domain, and large (L) that additionally contains the pre-S1 domain [117]. The L glycoprotein determines entry through its N-terminal pre-S1 domain and infection can be prevented through competition with a pre-S1 peptide [118,119,120]. NTCP, a liver BA transporter, was identified as a receptor for HBV using a synthetic photocrosslinkable pre-S1 peptide and mass spectrometry in tree shrew (*Tupaia belangeri*) primary hepatocytes [117]. Huh7 cells transfected with human NTCP show increased binding of HDV and HBV S1. Liver cancer cell lines have lost NTCP expression, but HepG2 cells that stably express NTCP are susceptible to HBV and HDV replication [117].

DMSO is known to differentiate many cell types, and a month-long presence of DMSO during culture of human primary hepatocyte HepaRG cells will differentiate these liver cells and lead to some NTCP expression [121]. Interestingly, DMSO can enhance replication of HBV/HDV in HepG2 cells expressing human NTCP when added during or shortly after infection indicating direct effects such as acting as a co-factor for entry or regulating the trafficking of NTCP [122]. An inhibitor of NTCP, Myrcludex B (MyrB), is a myristoylated synthetic lipopeptide of 47 amino acids of the HBV pre-S1 peptide, and it inhibits infection of HBV and TCA uptake. Treatment with multiple conjugated BAs (TCA/TDCA/TCDCA) also inhibits infection by HBV [122]. This effect was likely caused by BA blocking the binding of pre-S1 to NTCP as detection of labeled MyrB (pre-S1 peptide) on 500 µM BA-treated cells was reduced. Conversely, pre-S1 blocks labeled TCA uptake [117]. CA (minimal effect), TCA, GCA, LCA, DCA, TLCA, CDCA, UDCA, TUDCA, and hydrodeoxycholic (HDCA) can all block HBV and HDV infection of HepG2 NTCP cells, but not if added after inoculation, confirming the inhibitory effect is on viral entry through NTCP [117]. A region of human NTCP at amino acids 157–165 is critical for HBV infection and MyrB binding [122]. Together, these results indicate the HBV, MyrB, and BA binding sites overlap. Conflicting with these results is an HBV replicon system in HepG2 cells; CDCA treatment increases viral gene expression, although this system bypasses receptor binding and entry steps [123]. Similar to the HCV replicon system described above where viral entry is also bypassed, FXR antagonists (Z-guggulsterone and siRNA targeting FXR) in the presence of CDCA reduced HBV transcription [123].

NTCP overexpression in Huh7.5.1 cells also enhances HCV infection, but the mechanism is distinct from the enhancement of HBV/HDV infection because blocking the NTCP transporter with HBV pre-S1 1 h pretreatment does not affect HCV entry [124]. Longer pretreatment (24–72 h prior to infection) of cells with preS1 leads to the induction of interferon-stimulated genes including IFITM2 and IFITM3 and overexpression of these genes inhibits HCV replication [124]. When NTCP transports BAs into cells, IFITM2 and IFITM3 induction is suppressed and HCV can enter; therefore, pre-S1 blocking of NTCP modulates HCV entry by preventing BA-mediated suppression of the innate immune response.

In porcine kidney cells that support PoSaV infection in the presence of GCDCA, siRNA targeting the BA transporters NTCP and ASBT, but not FXR, reduces GCDCA-mediated PoSaV infection (Figure 5C) [67]. Detection of PoSaV levels at 1 hour post infection showed no changes in viral RNA levels in the presence of NTCP or ASBT siRNA, indicating that unlike the HBV model, inhibitory effects are not through a receptor binding effect [67]. NTCP and ASBT inhibitors have not yet been tested in HuNoV infection of HIEs from the jejunum or other segments of the small intestine for their effect on GII.3 replication. RNA sequencing data show that the presence of NTCP and ASBT is low (fragments per kilobase of transcript per million mapped reads is below 1 in jejunal HIEs) [1,108]. GII.3 can also replicate in the presence of GCDCA in duodenal and ileal lines and the ileal line has much higher ASBT expression. Future experiments are necessary to test if NTCP or ASBT influence HuNoV infection in HIEs from non-jejunal intestinal segments. Unlike the HBV system where NTCP is the cellular receptor for the virus, NTCP could still modulate levels of sapovirus or norovirus infection in the ileum through downstream cellular effects influenced by BA transport.

## 6. Conclusions and Future Perspectives

Viruses are unique engineers that, through evolution, exploit host cellular functions for the most effective replication tools. Noroviruses and hepatitis viruses have utilized host enterohepatic circulation factors (signaling molecules, receptors) to allow their entry and replication in different human cells (summarized in Table 3). Mechanistic studies of these gastrointestinal viruses are not only defining requirements for their replication but also providing new insights into human biology. Many experiments with a BA-dependent HuNoV were all performed in jejunal HIEs. Interestingly, we discovered that in jejunal HIEs, GCDCA acts through S1PR2, which has only recently been described to be activated by BAs in the liver and intestine to promote uptake and viral infection. The majority of studies of BA uptake and enterohepatic signaling pathways focus on the ileal segment of the small intestine. Our new results prompt further studies on how BAs communicate with jejunal cells to signal for endocytosis not only in the context of viral infection but for potential roles in human nutrient uptake.

Currently, there are no approved therapeutics for HuNoV infection. The BA sequestrant, cholestyramine, which removes BA from bile and therefore prevents GII.3 replication in HIEs, is an FDA-approved drug used to treat high cholesterol with limited side effects. Cholestyramine or the related drug colesevelam have potential for testing as therapeutics for those who develop chronic HuNoV infections. Furthermore, the ASM inhibitor, amitriptyline, is approved for use as an antidepressant. The shared requirement for ASM for both BA-dependent and independent HuNoVs as well as other caliciviruses (PoSaV, FCV, and MNV) makes this a candidate to test as a therapeutic for multiple strains of HuNoV infection. Inhibitors such as MyrB are being investigated for the potential to target NTCP for the development of HBV therapeutics [125]. These potential therapeutic targets, which were able to be discovered due to establishment of cultivation systems, highlight the importance of fully understanding the role enterohepatic circulation plays in mechanisms of viral infection. As cultivation systems for viruses that infect the gastrointestinal system continue to advance, so will our understanding of the human biology of nutrient uptake in the small intestine and our potential to treat these diseases.

## Figures and Tables

**Table 1:** Bile acid receptors.

Receptor Name	Relevant Tissue/Cellular Location
Apical sodium-dependent bile acid transporter (ASBT)	Ileal/membrane
Na^+^-taurocholate cotransporting polypeptide (NTCP)	Liver/basolateral membrane
Farnesoid X receptor (FXR)	Liver and intestine/intracellular
Sphingosine-1-phosphate receptor 2 (S1PR2)	Liver and intestine/membrane

**Table 2:** Effect of BA treatment on VLP * binding to PGM.

Genotype	Accession Number	GCDCA Effect on Binding
GI.1	M87661	No effect
GI.2	FJ515294	No effect
GI.4	GQ413970	No effect
GI.5	KJ402295	No effect
GI.6	KC998959	No effect
GI.7	JN005886	No effect
GI.8	GU299761	No binding +/−GCDCA
GII.1	JN797508	GCDCA permits binding
GII.2	AY134748	GCDCA permits binding
GII.3_2002	KF006265	No binding +/−GCDCA
GII.3_2004 **	AB365435	No effect
GII.4_Sydney	JX459908	No effect
GII.5	AF414422	No effect
GII.6	GU930737	No effect
GII.7	KF006266	No effect
GII.8	AB039780	No effect
GII.9	DQ379715	No effect
GII.10	AF504671	No binding +/−GCDCA
GII.12	KF006267	GCDCA permits binding
GII.13	JN899242	Enhancement of binding
GII.14	GU594162	Enhancement of binding
GII.15	GQ856474	No binding +/−GCDCA
GII.17	DQ438972	No effect

* VLPs generated from baculovirus-expressed ORF2 + 3 + 3**′**UTR. Previously unpublished data except for GII.3_2004 (**) published in Murakami et al., 2020 [1]. The PGM binding assays are performed as described in Murakami et al., 2020 [1].

**Table 3:** Summary of BA effects on viral infection.

Virus	Required for Infection	STD-NMR Binding Site	ITC Binding Site	Required for HBGA Binding	Mediates Suppression of IFN Response	Other Effects		
GI.1 HuNoV	+	+			+			
	HIEs				(replicon only)			
GII.1 HuNoV	+		+	+				
	HIEs							
GII.2 HuNoV				+				
GII.3 HuNoV	+					BAs mediate cellular effects (e.g., increased uptake, ASM activity, and ceramide generation)		
	HIEs							
GII.4 HuNoV	BAs enhance infection of HIEs	+				Requires ASM activity		
GII.6 HuNoV	+							
	HIEs							
GII.7 HuNoV		+						
GII.10 HuNoV		+	+	+				
GII.12 HuNoV				+				
GII.13 HuNoV				BA enhances binding				
GII.14 HuNoV				BA enhances binding				
GII.17 HuNoV	+	+						
	HIEs							
GII.19 HuNoV		+						
PoSaV	+					BA mediates cellular effects (e.g., endosomal escape, ASM activity, and ceramide generation)		
	LLC-PK							
HuSaV	+							
	NEC8 and HuTu80							
MNV	BAs enhance infection BV-2 and distal GALT				+	Requires ASM activity, BA binds P-domain at the dimer interface, BA enhances binding to the CD300lf receptor		
FCV						Requires ASM activity		
HAV						BAs disrupt enveloped particle		
HBV						Cellular receptor is BA receptor, NTCP		
HCV					+			
HEV						BAs disrupt enveloped particle		

## Data Availability

All data are present in the main text or available from the authors upon request.

## References

[1] Murakami K., Tenge V.R., Karandikar U.C., Lin S.C., Ramani S., Ettayebi K., Crawford S.E., Zeng X.L., Neill F.H., Ayyar B.V. (2020). Bile acids and ceramide overcome the entry restriction for GII.3 human norovirus replication in human intestinal enteroids. Proc. Natl. Acad. Sci. USA.

[2] Yu Y., Li S., Liang W. (2018). Bona fide receptor for hepatitis B and D viral infections: Mechanism, research models and molecular drug targets. Emerg. Microbes Infect..

[3] Chang K.O., George D.W. (2007). Bile acids promote the expression of hepatitis C virus in replicon-harboring cells. J. Virol..

[4] Grau K.R., Zhu S., Peterson S.T., Helm E.W., Philip D., Phillips M., Hernandez A., Turula H., Frasse P., Graziano V.R. (2020). The intestinal regionalization of acute norovirus infection is regulated by the microbiota via bile acid-mediated priming of type III interferon. Nat. Microbiol..

[5] Barrett K.E., Barman S.M., Brooks H.L., Yuan J.X.J. (2019). Overview of Gastrointestinal Function & Regulation. Ganong’s Review of Medical Physiology, 26e.

[6] Kuipers F., Bloks V.W., Groen A.K. (2014). Beyond intestinal soap—Bile acids in metabolic control. Nat. Rev. Endocrinol..

[7] Jones M.W., Hannoodee S., Young M. (2021). Anatomy, Abdomen and Pelvis, Gallbladder. StatPearls.

[8] Boyer J.L. (2013). Bile formation and secretion. Compr. Physiol..

[9] Chiang J.Y.L. (2009). Bile acids: Regulation of synthesis. J. Lipid Res..

[10] Chiang J.Y. (2013). Bile acid metabolism and signaling. Compr. Physiol..

[11] Daruich A., Picard E., Boatright J.H., Behar-Cohen F. (2019). Review: The bile acids urso- and tauroursodeoxycholic acid as neuroprotective therapies in retinal disease. Mol. Vis..

[12] Ridlon J.M., Bajaj J.S. (2015). The human gut sterolbiome: Bile acid-microbiome endocrine aspects and therapeutics. Acta Pharm. Sin. B.

[13] Hegyi P., Maléth J., Walters J.R., Hofmann A.F., Keely S.J. (2018). Guts and Gall: Bile Acids in Regulation of Intestinal Epithelial Function in Health and Disease. Physiol. Rev..

[14] Keely S.J., Steer C.J., Lajczak-McGinley N.K. (2019). Ursodeoxycholic acid: A promising therapeutic target for inflammatory bowel diseases?. Am. J. Physiol. Gastrointest. Liver Physiol..

[15] Dawson P.A., Said H.M. (2018). Chapter 41—Bile Formation and the Enterohepatic Circulation. Physiology of the Gastrointestinal Tract.

[16] Matsubara T., Li F., Gonzalez F.J. (2013). FXR signaling in the enterohepatic system. Mol. Cell Endocrinol..

[17] Fiorucci S., Biagioli M., Zampella A., Distrutti E. (2018). Bile Acids Activated Receptors Regulate Innate Immunity. Front. Immunol..

[18] Northfield T.C., McColl I. (1973). Postprandial concentrations of free and conjugated bile acids down the length of the normal human small intestine. Gut.

[19] Van Deest B.W., Fordtran J.S., Morawski S.G., Wilson J.D. (1968). Bile salt and micellar fat concentration in proximal small bowel contents of ileectomy patients. J. Clin. Investig..

[20] Martinez-Augustin O., Sanchez de Medina F. (2008). Intestinal bile acid physiology and pathophysiology. World J. Gastroenterol..

[21] Hofmann A.F., Eckmann L. (2006). How bile acids confer gut mucosal protection against bacteria. Proc. Natl. Acad. Sci. USA.

[22] Ahmed S.M., Hall A.J., Robinson A.E., Verhoef L., Premkumar P., Parashar U.D., Koopmans M., Lopman B.A. (2014). Global prevalence of norovirus in cases of gastroenteritis: A systematic review and meta-analysis. Lancet Infect. Dis..

[23] Hall A.J., Lopman B.A., Payne D.C., Patel M.M., Gastanaduy P.A., Vinje J., Parashar U.D. (2013). Norovirus disease in the United States. Emerg. Infect. Dis..

[24] Pires S.M., Fischer-Walker C.L., Lanata C.F., Devleesschauwer B., Hall A.J., Kirk M.D., Duarte A.S., Black R.E., Angulo F.J. (2015). Aetiology-specific estimates of the global and regional incidence and mortality of diarrhoeal diseases commonly transmitted through food. PLoS ONE.

[25] Ramani S., Atmar R.L., Estes M.K. (2014). Epidemiology of human noroviruses and updates on vaccine development. Curr. Opin. Gastroenterol..

[26] Havelaar A.H., Kirk M.D., Torgerson P.R., Gibb H.J., Hald T., Lake R.J., Praet N., Bellinger D.C., de Silva N.R., Gargouri N. (2015). World Health Organization global estimates and regional comparisons of the burden of foodborne disease in 2010. PLoS Med..

[27] Belliot G., Lopman B.A., Ambert-Balay K., Pothier P. (2014). The burden of norovirus gastroenteritis: An important foodborne and healthcare-related infection. Clin. Microbiol. Infect..

[28] Bartsch S.M., Lopman B.A., Ozawa S., Hall A.J., Lee B.Y. (2016). Global economic burden of norovirus gastroenteritis. PLoS ONE.

[29] Kapikian A.Z., Wyatt R.G., Dolin R., Thornhill T.S., Kalica A.R., Chanock R.M. (1972). Visualization by immune electron microscopy of a 27-nm particle associated with acute infectious nonbacterial gastroenteritis. J. Virol..

[30] Adler J.L., Zickl R. (1969). Winter Vomiting Disease. J. Infect. Dis..

[31] Duizer E., Schwab K.J., Neill F.H., Atmar R.L., Koopmans M.P., Estes M.K. (2004). Laboratory efforts to cultivate noroviruses. J. Gen. Virol..

[32] Lay M.K., Atmar R.L., Guix S., Bharadwaj U., He H., Neill F.H., Sastry K.J., Yao Q., Estes M.K. (2010). Norwalk virus does not replicate in human macrophages or dendritic cells derived from the peripheral blood of susceptible humans. Virology.

[33] Herbst-Kralovetz M.M., Radtke A.L., Lay M.K., Hjelm B.E., Bolick A.N., Sarker S.S., Atmar R.L., Kingsley D.H., Arntzen C.J., Estes M.K. (2013). Lack of norovirus replication and histo-blood group antigen expression in 3-dimensional intestinal epithelial cells. Emerg. Infect. Dis..

[34] Papafragkou E., Hewitt J., Park G.W., Greening G., Vinje J. (2014). Challenges of culturing human norovirus in three-dimensional organoid intestinal cell culture models. PLoS ONE.

[35] Straub T.M., Bartholomew R.A., Valdez C.O., Valentine N.B., Dohnalkova A., Ozanich R.M., Bruckner-Lea C.J., Call D.R. (2011). Human norovirus infection of caco-2 cells grown as a three-dimensional tissue structure. J. Water Health.

[36] Straub T.M., Höner zu Bentrup K., Orosz-Coghlan P., Dohnalkova A., Mayer B.K., Bartholomew R.A., Valdez C.O., Bruckner-Lea C.J., Gerba C.P., Abbaszadegan M. (2007). In vitro cell culture infectivity assay for human noroviruses. Emerg. Infect. Dis..

[37] Straub T.M., Hutchison J.R., Bartholomew R.A., Valdez C.O., Valentine N.B., Dohnalkova A., Ozanich R.M., Bruckner-Lea C.J. (2013). Defining cell culture conditions to improve human norovirus infectivity assays. Water Sci. Technol..

[38] Takanashi S., Saif L.J., Hughes J.H., Meulia T., Jung K., Scheuer K.A., Wang Q. (2014). Failure of propagation of human norovirus in intestinal epithelial cells with microvilli grown in three-dimensional cultures. Arch. Virol..

[39] Huang P., Farkas T., Marionneau S., Zhong W., Ruvoen-Clouet N., Morrow A.L., Altaye M., Pickering L.K., Newburg D.S., LePendu J. (2003). Noroviruses bind to human ABO, Lewis, and secretor histo-blood group antigens: Identification of 4 distinct strain-specific patterns. J. Infect. Dis..

[40] Marionneau S., Ruvoën N., Le Moullac-Vaidye B., Clement M., Cailleau-Thomas A., Ruiz-Palacois G., Huang P., Jiang X., Le Pendu J. (2002). Norwalk virus binds to histo-blood group antigens present on gastroduodenal epithelial cells of secretor individuals. Gastroenterology.

[41] Harrington P.R., Lindesmith L., Yount B., Moe C.L., Baric R.S. (2002). Binding of Norwalk virus-like particles to ABH histo-blood group antigens is blocked by antisera from infected human volunteers or experimentally vaccinated mice. J. Virol..

[42] Hutson A.M., Atmar R.L., Marcus D.M., Estes M.K. (2003). Norwalk virus-like particle hemagglutination by binding to h histo-blood group antigens. J. Virol..

[43] Choi J.M., Hutson A.M., Estes M.K., Prasad B.V. (2008). Atomic resolution structural characterization of recognition of histo-blood group antigens by Norwalk virus. Proc. Natl. Acad. Sci. USA.

[44] Singh B.K., Leuthold M.M., Hansman G.S. (2015). Human Noroviruses’ Fondness for Histo-Blood Group Antigens. J. Virol..

[45] Weichert S., Koromyslova A., Singh B.K., Hansman S., Jennewein S., Schroten H., Hansman G.S. (2016). Structural Basis for Norovirus Inhibition by Human Milk Oligosaccharides. J. Virol..

[46] Lindesmith L., Moe C., Marionneau S., Ruvoen N., Jiang X., Lindblad L., Stewart P., LePendu J., Baric R. (2003). Human susceptibility and resistance to Norwalk virus infection. Nat. Med..

[47] Hutson A.M., Airaud F., LePendu J., Estes M.K., Atmar R.L. (2005). Norwalk virus infection associates with secretor status genotyped from sera. J. Med. Virol..

[48] Hutson A.M., Atmar R.L., Graham D.Y., Estes M.K. (2002). Norwalk virus infection and disease is associated with ABO histo-blood group type. J. Infect. Dis..

[49] Tan M., Xia M., Chen Y., Bu W., Hegde R.S., Meller J., Li X., Jiang X. (2009). Conservation of carbohydrate binding interfaces: Evidence of human HBGA selection in norovirus evolution. PLoS ONE.

[50] Guix S., Asanaka M., Katayama K., Crawford S.E., Neill F.H., Atmar R.L., Estes M.K. (2007). Norwalk virus RNA is infectious in mammalian cells. J. Virol..

[51] Ettayebi K., Crawford S.E., Murakami K., Broughman J.R., Karandikar U., Tenge V.R., Neill F.H., Blutt S.E., Zeng X.L., Qu L. (2016). Replication of human noroviruses in stem cell-derived human enteroids. Science.

[52] Estes M.K., Ettayebi K., Tenge V.R., Murakami K., Karandikar U., Lin S.C., Ayyar B.V., Cortes-Penfield N.W., Haga K., Neill F.H. (2019). Human Norovirus Cultivation in Nontransformed Stem Cell-Derived Human Intestinal Enteroid Cultures: Success and Challenges. Viruses.

[53] Ettayebi K., Tenge V.R., Cortes-Penfield N.W., Crawford S.E., Neill F.H., Zeng X.-L., Yu X., Ayyar B.V., Burrin D., Ramani S. (2021). New Insights and Enhanced Human Norovirus Cultivation in Human Intestinal Enteroids. mSphere.

[54] Wilen C.B., Lee S., Hsieh L.L., Orchard R.C., Desai C., Hykes B.L., McAllaster M.R., Balce D.R., Feehley T., Brestoff J.R. (2018). Tropism for tuft cells determines immune promotion of norovirus pathogenesis. Science.

[55] Wobus C.E., Karst S.M., Thackray L.B., Chang K.O., Sosnovtsev S.V., Belliot G., Krug A., Mackenzie J.M., Green K.Y., Virgin H.W. (2004). Replication of Norovirus in cell culture reveals a tropism for dendritic cells and macrophages. PLoS Biol..

[56] Jones M.K., Watanabe M., Zhu S., Graves C.L., Keyes L.R., Grau K.R., Gonzalez-Hernandez M.B., Iovine N.M., Wobus C.E., Vinje J. (2014). Enteric bacteria promote human and mouse norovirus infection of B cells. Science.

[57] Flynn W.T., Saif L.J. (1988). Serial propagation of porcine enteric calicivirus-like virus in primary porcine kidney cell cultures. J. Clin. Microbiol..

[58] Parwani A.V., Flynn W.T., Gadfield K.L., Saif L.J. (1991). Serial propagation of porcine enteric calicivirus in a continuous cell line. Effect of medium supplementation with intestinal contents or enzymes. Arch. Virol..

[59] Makino A., Shimojima M., Miyazawa T., Kato K., Tohya Y., Akashi H. (2006). Junctional adhesion molecule 1 is a functional receptor for feline calicivirus. J. Virol..

[60] Ossiboff R.J., Parker J.S. (2007). Identification of regions and residues in feline junctional adhesion molecule required for feline calicivirus binding and infection. J. Virol..

[61] Alfajaro M.M., Cho E.H., Kim D.S., Kim J.Y., Park J.G., Soliman M., Baek Y.B., Park C.H., Kang M.I., Park S.I. (2019). Early porcine sapovirus infection disrupts tight junctions and uses occludin as a coreceptor. J. Virol..

[62] Orchard R.C., Wilen C.B., Doench J.G., Baldridge M.T., McCune B.T., Lee Y.C., Lee S., Pruett-Miller S.M., Nelson C.A., Fremont D.H. (2016). Discovery of a proteinaceous cellular receptor for a norovirus. Science.

[63] Nagai M., Wang Q., Oka T., Saif L.J. (2020). Porcine sapoviruses: Pathogenesis, epidemiology, genetic diversity, and diagnosis. Virus Res..

[64] Karst S.M., Wobus C.E., Lay M., Davidson J., Virgin H.W. (2003). STAT1-dependent innate immunity to a Norwalk-like virus. Science.

[65] Graziano V.R., Walker F.C., Kennedy E.A., Wei J., Ettayebi K., Strine M.S., Filler R.B., Hassan E., Hsieh L.L., Kim A.S. (2020). CD300lf is the primary physiologic receptor of murine norovirus but not human norovirus. PLoS Pathog..

[66] Chang K.O., Sosnovtsev S.V., Belliot G., Kim Y., Saif L.J., Green K.Y. (2004). Bile acids are essential for porcine enteric calicivirus replication in association with down-regulation of signal transducer and activator of transcription 1. Proc. Natl. Acad. Sci. USA.

[67] Shivanna V., Kim Y., Chang K.O. (2014). The crucial role of bile acids in the entry of porcine enteric calicivirus. Virology.

[68] Shivanna V., Kim Y., Chang K.O. (2014). Endosomal acidification and cathepsin L activity is required for calicivirus replication. Virology.

[69] Takagi H., Oka T., Shimoike T., Saito H., Kobayashi T., Takahashi T., Tatsumi C., Kataoka M., Wang Q., Saif L.J. (2020). Human sapovirus propagation in human cell lines supplemented with bile acids. Proc. Natl. Acad. Sci. USA.

[70] Shivanna V., Kim Y., Chang K.O. (2015). Ceramide formation mediated by acid sphingomyelinase facilitates endosomal escape of caliciviruses. Virology.

[71] Contreras F.X., Sanchez-Magraner L., Alonso A., Goni F.M. (2010). Transbilayer (flip-flop) lipid motion and lipid scrambling in membranes. FEBS Lett..

[72] Ruiz-Argüello M.B., Basáñez G., Goñi F.M., Alonso A. (1996). Different effects of enzyme-generated ceramides and diacylglycerols in phospholipid membrane fusion and leakage. J. Biol. Chem..

[73] Gulbins E., Dreschers S., Wilker B., Grassmé H. (2004). Ceramide, membrane rafts and infections. J. Mol. Med..

[74] Luisoni S., Suomalainen M., Boucke K., Tanner L.B., Wenk M.R., Guan X.L., Grzybek M., Coskun U., Greber U.F. (2015). Co-option of Membrane Wounding Enables Virus Penetration into Cells. Cell Host Microbe.

[75] Avota E., Gulbins E., Schneider-Schaulies S. (2011). DC-SIGN mediated sphingomyelinase-activation and ceramide generation is essential for enhancement of viral uptake in dendritic cells. PLoS Pathog..

[76] Grassmé H., Riehle A., Wilker B., Gulbins E. (2005). Rhinoviruses infect human epithelial cells via ceramide-enriched membrane platforms. J. Biol. Chem..

[77] Tani H., Shiokawa M., Kaname Y., Kambara H., Mori Y., Abe T., Moriishi K., Matsuura Y. (2010). Involvement of ceramide in the propagation of Japanese encephalitis virus. J. Virol..

[78] Miller M.E., Adhikary S., Kolokoltsov A.A., Davey R.A. (2012). Ebolavirus requires acid sphingomyelinase activity and plasma membrane sphingomyelin for infection. J. Virol..

[79] Holly M.K., Smith J.G. (2018). Adenovirus Infection of Human Enteroids Reveals Interferon Sensitivity and Preferential Infection of Goblet Cells. J. Virol..

[80] Conley M.J., McElwee M., Azmi L., Gabrielsen M., Byron O., Goodfellow I.G., Bhella D. (2019). Calicivirus VP2 forms a portal-like assembly following receptor engagement. Nature.

[81] Kilic T., Koromyslova A., Hansman G.S. (2019). Structural basis for human norovirus capsid binding to bile acids. J. Virol..

[82] Creutznacher R., Schulze E., Wallmann G., Peters T., Stein M., Mallagaray A. (2020). Chemical-Shift Perturbations Reflect Bile Acid Binding to Norovirus Coat Protein: Recognition Comes in Different Flavors. Chembiochem.

[83] Costantini V., Morantz E.K., Browne H., Ettayebi K., Zeng X.L., Atmar R.L., Estes M.K., Vinje J. (2018). Human norovirus replication in human intestinal enteroids as model to evaluate virus inactivation. Emerg. Infect. Dis..

[84] Lindesmith L.C., Brewer-Jensen P.D., Mallory M.L., Jensen K., Yount B.L., Costantini V., Collins M.H., Edwards C.E., Sheahan T.P., Vinjé J. (2020). Virus-Host Interactions Between Nonsecretors and Human Norovirus. Cell. Mol. Gastroenterol. Hepatol..

[85] Graziano V.R., Alfajaro M.M., Schmitz C.O., Filler R.B., Strine M.S., Wei J., Hsieh L.L., Baldridge M.T., Nice T.J., Lee S. (2021). CD300lf Conditional Knockout Mouse Reveals Strain-Specific Cellular Tropism of Murine Norovirus. J. Virol..

[86] Nelson C.A., Wilen C.B., Dai Y.N., Orchard R.C., Kim A.S., Stegeman R.A., Hsieh L.L., Smith T.J., Virgin H.W., Fremont D.H. (2018). Structural basis for murine norovirus engagement of bile acids and the CD300lf receptor. Proc. Natl. Acad. Sci. USA.

[87] Smith H.Q., Smith T.J. (2019). The Dynamic Capsid Structures of the Noroviruses. Viruses.

[88] Sherman M.B., Smith H.Q., Smith T.J. (2020). The Dynamic Life of Virus Capsids. Viruses.

[89] Sherman M.B., Williams A.N., Smith H.Q., Nelson C., Wilen C.B., Fremont D.H., Virgin H.W., Smith T.J. (2019). Bile Salts Alter the Mouse Norovirus Capsid Conformation: Possible Implications for Cell Attachment and Immune Evasion. J. Virol..

[90] Williams A.N., Sherman M.B., Smith H.Q., Taube S., Pettitt B.M., Wobus C.E., Smith T.J. (2021). A norovirus uses bile salts to escape antibody recognition while enhancing receptor binding. J. Virol..

[91] Song C., Takai-Todaka R., Miki M., Haga K., Fujimoto A., Ishiyama R., Oikawa K., Yokoyama M., Miyazaki N., Iwasaki K. (2020). Dynamic rotation of the protruding domain enhances the infectivity of norovirus. PLOS Pathog..

[92] Di Ciaula A., Garruti G., Lunardi Baccetto R., Molina-Molina E., Bonfrate L., Wang D.Q., Portincasa P. (2017). Bile Acid Physiology. Ann. Hepatol..

[93] Ridlon J.M., Kang D.J., Hylemon P.B., Bajaj J.S. (2014). Bile acids and the gut microbiome. Curr. Opin. Gastroenterol..

[94] Dossa A.Y., Escobar O., Golden J., Frey M.R., Ford H.R., Gayer C.P. (2016). Bile acids regulate intestinal cell proliferation by modulating EGFR and FXR signaling. Am. J. Physiol. Gastrointest. Liver Physiol..

[95] Zarrin A., Akhondi H. (2020). Viral Hepatitis. StatPearls.

[96] Manns M.P., Buti M., Gane E., Pawlotsky J.M., Razavi H., Terrault N., Younossi Z. (2017). Hepatitis C virus infection. Nat. Rev. Dis. Primers.

[97] Lohmann V., Bartenschlager R. (2014). On the history of hepatitis C virus cell culture systems. J. Med. Chem..

[98] Wakita T., Pietschmann T., Kato T., Date T., Miyamoto M., Zhao Z., Murthy K., Habermann A., Kräusslich H.G., Mizokami M. (2005). Production of infectious hepatitis C virus in tissue culture from a cloned viral genome. Nat. Med..

[99] Zhong J., Gastaminza P., Cheng G., Kapadia S., Kato T., Burton D.R., Wieland S.F., Uprichard S.L., Wakita T., Chisari F.V. (2005). Robust hepatitis C virus infection in vitro. Proc. Natl. Acad. Sci. USA.

[100] Lindenbach B.D., Evans M.J., Syder A.J., Wölk B., Tellinghuisen T.L., Liu C.C., Maruyama T., Hynes R.O., Burton D.R., McKeating J.A. (2005). Complete replication of hepatitis C virus in cell culture. Science.

[101] Wakita T., Tang H. (2009). Isolation of JFH-1 Strain and Development of an HCV Infection System. Hepatitis C: Methods and Protocols.

[102] Scholtes C., Diaz O., Icard V., Kaul A., Bartenschlager R., Lotteau V., Andre P. (2008). Enhancement of genotype 1 hepatitis C virus replication by bile acids through FXR. J. Hepatol..

[103] Chhatwal P., Bankwitz D., Gentzsch J., Frentzen A., Schult P., Lohmann V., Pietschmann T. (2012). Bile acids specifically increase hepatitis C virus RNA-replication. PLoS ONE.

[104] Kullak-ublick G.A., Stieger B., Meier P.J. (2004). Enterohepatic bile salt transporters in normal physiology and liver disease. Gastroenterology.

[105] Wang Y.-D., Chen W.-D., Moore D.D., Huang W. (2008). FXR: A metabolic regulator and cell protector. Cell Res..

[106] Qu L., Murakami K., Broughman J.R., Lay M.K., Guix S., Tenge V.R., Atmar R.L., Estes M.K. (2016). Replication of human norovirus RNA in mammalian cells reveals lack of interferon response. J. Virol..

[107] Hosmillo M., Sorgeloos F., Hiraide R., Lu J., Goodfellow I., Cho K.O. (2015). Porcine sapovirus replication is restricted by the type I interferon response in cell culture. J. Gen. Virol..

[108] Lin S.-C., Qu L., Ettayebi K., Crawford S.E., Blutt S.E., Robertson M.J., Zeng X.-L., Tenge V.R., Ayyar B.V., Karandikar U.C. (2020). Human norovirus exhibits strain-specific sensitivity to host interferon pathways in human intestinal enteroids. Proc. Natl. Acad. Sci. USA.

[109] Feng Z., Hensley L., McKnight K.L., Hu F., Madden V., Ping L., Jeong S.H., Walker C., Lanford R.E., Lemon S.M. (2013). A pathogenic picornavirus acquires an envelope by hijacking cellular membranes. Nature.

[110] Hirai-Yuki A., Hensley L., Whitmire J.K., Lemon S.M. (2016). Biliary Secretion of Quasi-Enveloped Human Hepatitis A Virus. mBio.

[111] Takahashi M., Tanaka T., Takahashi H., Hoshino Y., Nagashima S., Jirintai, Mizuo H., Yazaki Y., Takagi T., Azuma M. (2010). Hepatitis E Virus (HEV) strains in serum samples can replicate efficiently in cultured cells despite the coexistence of HEV antibodies: Characterization of HEV virions in blood circulation. J. Clin. Microbiol..

[112] Yin X., Li X., Feng Z. (2016). Role of Envelopment in the HEV Life Cycle. Viruses.

[113] Sayed I.M., Verhoye L., Cocquerel L., Abravanel F., Foquet L., Montpellier C., Debing Y., Farhoudi A., Wychowski C., Dubuisson J. (2017). Study of hepatitis E virus infection of genotype 1 and 3 in mice with humanised liver. Gut.

[114] Santiana M., Ghosh S., Ho B.A., Rajasekaran V., Du W.L., Mutsafi Y., De Jesus-Diaz D.A., Sosnovtsev S.V., Levenson E.A., Parra G.I. (2018). Vesicle-cloaked virus clusters are optimal units for inter-organismal viral transmission. Cell Host Microbe.

[115] Mentha N., Clément S., Negro F., Alfaiate D. (2019). A review on hepatitis D: From virology to new therapies. J. Adv. Res..

[116] Hu J., Lin Y.Y., Chen P.J., Watashi K., Wakita T. (2019). Cell and Animal Models for Studying Hepatitis B Virus Infection and Drug Development. Gastroenterology.

[117] Yan H., Zhong G., Xu G., He W., Jing Z., Gao Z., Huang Y., Qi Y., Peng B., Wang H. (2012). Sodium taurocholate cotransporting polypeptide is a functional receptor for human hepatitis B and D virus. eLife.

[118] Barrera A., Guerra B., Notvall L., Lanford R.E. (2005). Mapping of the hepatitis B virus pre-S1 domain involved in receptor recognition. J. Virol..

[119] Glebe D., Urban S., Knoop E.V., Cag N., Krass P., Grün S., Bulavaite A., Sasnauskas K., Gerlich W.H. (2005). Mapping of the hepatitis B virus attachment site by use of infection-inhibiting preS1 lipopeptides and tupaia hepatocytes. Gastroenterology.

[120] Engelke M., Mills K., Seitz S., Simon P., Gripon P., Schnölzer M., Urban S. (2006). Characterization of a hepatitis B and hepatitis delta virus receptor binding site. Hepatology.

[121] Kotani N., Maeda K., Debori Y., Camus S., Li R., Chesne C., Sugiyama Y. (2012). Expression and Transport Function of Drug Uptake Transporters in Differentiated HepaRG Cells. Mol. Pharm..

[122] Ni Y., Lempp F.A., Mehrle S., Nkongolo S., Kaufman C., Fälth M., Stindt J., Königer C., Nassal M., Kubitz R. (2014). Hepatitis B and D viruses exploit sodium taurocholate co-transporting polypeptide for species-specific entry into hepatocytes. Gastroenterology.

[123] Kim H.Y., Cho H.K., Choi Y.H., Lee K.S., Cheong J. (2010). Bile acids increase hepatitis B virus gene expression and inhibit interferon-alpha activity. FEBS J..

[124] Verrier E.R., Colpitts C.C., Bach C., Heydmann L., Zona L., Xiao F., Thumann C., Crouchet E., Gaudin R., Sureau C. (2016). Solute Carrier NTCP Regulates Innate Antiviral Immune Responses Targeting Hepatitis C Virus Infection of Hepatocytes. Cell Rep..

[125] Donkers J.M., Zehnder B., van Westen G.J.P., Kwakkenbos M.J., AP I.J., Oude Elferink R.P.J., Beuers U., Urban S., van de Graaf S.F.J. (2017). Reduced hepatitis B and D viral entry using clinically applied drugs as novel inhibitors of the bile acid transporter NTCP. Sci. Rep..

